# Celiac Disease, Gluten-Free Diet, and Eating Disorders: From Bench to Bedside

**DOI:** 10.3390/foods14010074

**Published:** 2024-12-31

**Authors:** Yaohui Wei, Yating Wang, Yonggui Yuan, Jue Chen

**Affiliations:** 1 Department of Clinical Psychology, Shanghai Mental Health Center, Shanghai Jiao Tong University School of Medicine, Shanghai 200030, China; weiyaohui0108@gmail.com (Y.W.); wangyating@sjtu.edu.cn (Y.W.); 2 Department of Psychiatry and Psychotherapy, Klinikum rechts der Isar, Technical University of Munich, 81675 Munich, Germany; 3 Department of Psychosomatics and Psychiatry, Zhongda Hospital, School of Medicine, Jiangsu Provincial Key Laboratory of Brain Science and Medicine, Southeast University, Nanjing 210009, China

**Keywords:** celiac disease, disordered eating behaviors, eating disorder, gluten-free diet

## Abstract

Celiac disease (CD) and eating disorders (EDs) are complex chronic conditions in adolescents, sharing symptoms such as weight change, malnutrition, and gastrointestinal symptoms. CD, an autoimmune disorder triggered by gluten ingestion, is managed through a strict gluten-free diet that can unintentionally foster disordered eating behaviors due to dietary restrictions. Conversely, EDs may mask and complicate CD symptoms, leading to diagnostic delays and treatment challenges. Evidence reveals an increased risk of EDs in CD individuals and vice versa, indicating a potential bidirectional relationship. This review explores the mechanisms and clinical implications of this interplay and proposes integrated screening and care strategies to improve the quality of life for individuals with both conditions.

## 1. Introduction

Celiac disease (CD) is one of the most common chronic autoimmune disorders, characterized by small intestinal villi atrophy and inflammation. It occurs in genetically predisposed individuals upon ingesting gluten, a protein found in wheat, rye, oats, and barley [1]. With a prevalence of 0.5% to 2% in the general population, it poses a notable global public health challenge [2].

Currently, a strict, life-long gluten-free diet (GFD) remains the only recommended treatment for CD, which usually alleviates symptoms and improves mucosal damage in the small intestine [3]. However, not all individuals adequately respond to the GFD, either symptomatically or histologically, mainly due to inadvertent gluten exposure [3,4]. GFD maintenance is challenging, particularly when eating food prepared by others outside the home, where cross-contamination can easily happen. Even naturally gluten-free grains can also become contaminated with gluten-containing grains during planting, harvesting, or processing stages [5]. Therefore, maintaining a GFD can be burdensome, leading to or exacerbating underlying tendencies for depression, anxiety, disordered eating, social isolation, and impaired overall quality of life [6,7,8,9].

Eating disorders (EDs) are serious psychiatric conditions, characterized by disordered eating behaviors, body image disturbances, and various physical and nutritional health issues [10]. According to its characteristics, EDs can be mainly divided into anorexia nervosa (AN), bulimia nervosa (BN), and binge eating disorder. A systematic review, published between 1994 and 2013, found the prevalence of EDs varied widely, ranging from 1.0% to 22.7% in women and from 0.3% to 0.6% in men [11]. The causes of EDs are so complex that treatment often involves multiple healthcare disciplines with modest results [12].

Recently, emerging evidence has shown that EDs may inextricably interact with CD. There is a significant bidirectional association between these two conditions. People with CD have a significantly higher risk of developing EDs than healthy people, and similarly, people with EDs have a significantly higher overall risk of developing CD [9,13,14,15]. EDs and CD are more common in women and are associated with a variety of mental disorders, such as attention-deficit/hyperactivity disorder, anxiety, and mood disorder [7,16,17,18]. This article reviews the bidirectional relationship and pathogenesis of CD and EDs to provide new insights into the management of both conditions.

## 2. Materials and Methods

This narrative review investigates the bidirectional relationship between CD and EDs, synthesizing evidence from clinical, psychological, and nutritional research. A systematic literature search was conducted in the PubMed and MEDLINE databases using keywords such as “celiac disease”, “eating disorders”, “anorexia nervosa”, “bulimia nervosa”, “binge eating disorder”, “gluten-free diet”, and “mental health”.

Inclusion criteria comprised observational studies, randomized controlled trials, retrospective studies, review articles, case reports, and other relevant articles that examined both CD and EDs. Studies were selected if they investigated the following: (1) the impact of a CD on ED symptoms or vice versa; (2) psychological and psychosocial factors related to both conditions; (3) the role of a gluten-free diet and nutritional deficiencies; and (4) quality of life in affected individuals.

Clinical studies focusing on the management of CD and EDs, including treatment strategies and psychological interventions, were also reviewed. Additional studies exploring neurobiological mechanisms, such as the gut–brain axis, immune dysregulation, and genetic predispositions, were included to provide a comprehensive understanding of the underlying connections between CD and Eds. This review integrates findings from diverse research fields to offer insights into the complex relationship between these two conditions and their clinical implications.

## 3. Bidirectional Relationships Between Celiac Disease and Eating Disorders

Symptoms of CD and Eds often overlap, with common features including weight change, malnutrition, and non-specific gastrointestinal distress (Table 1). An estimated majority of the 3 million Americans with CD remain undiagnosed, often because CD symptoms can be silent or subclinical [19]. Poor weight gain might be one of the classic initial presentations and is often followed by failure to thrive in an adolescent or young adult. Undiagnosed CD can lead to long-term health conditions and other autoimmune disorders, including chronic nutritional deficiencies, early onset osteoporosis or osteopenia, iron deficiency anemia, intestinal lymphoma, and neurological symptoms [20,21]. Given that these symptoms lack specificity and overlap with many symptoms of Eds, it is important to consider the possibilities of CD for any adolescent and young adult being evaluated for an eating disorder diagnosis, or vice versa.

### 3.1. Impact of Celiac Disease on Eating Disorders

CD can cause a range of gastrointestinal discomfort, including bloating, abdominal pain or cramping, feeling of fullness or heaviness in the stomach, and changes in bowel habits—symptoms that are frequently observed in various EDs. Individuals with CD have to maintain a GFD to avoid such gastrointestinal distress, requiring vigilance and control over food choices, which can mimic or trigger disordered eating patterns. For instance, individuals with CD may initially focus intensely on food intake to manage physical symptoms, but this can evolve into an excessive concern with other aspects of consumption, such as overall calorie count, as seen in conditions like AN and BN [22]. Psychological well-being and quality of life are particularly lower in CD women compared to their male counterparts; specifically, women experienced more bowel symptoms, found it harder to manage feelings of deprivation associated with a GFD, and focused more on controlling every meal, than did men with CD [23,24]. Cadenhead et al. [25] discovered that approximately half of the adolescents with CD employed maladaptive approaches to maintaining a GFD, similar to known risk factors for feeding and EDs, such as rigidity, avoidance, controlling behaviors, and an excessive focus on food. Other disordered eating behaviors observed in individuals with CD include dieting for shape and weight control, excessive exercise, vomiting, laxative overuse, and binge eating [26,27].

Studies have demonstrated a high prevalence of EDs among individuals diagnosed with CD, especially females. A 2021 meta-analysis described the significant association between CD and EDs, in both the prevalence of EDs in individuals with CD and vice-versa [13]. The investigators found that the pooled prevalence of EDs in individuals with CD was 8.88%, with subgroup meta-analysis revealing a rate of 6.37% in adults and 11.97% in children, and the prevalence of BN was 7.26% [13]. These values exceed those documented in previous systematic reviews of the general population [28,29,30], indicating that the development of CD might intersect with factors that precipitate EDs.

### 3.2. Impact of Eating Disorders on Celiac Disease

Conversely, the presence of an eating disorder can complicate the diagnosis of CD. Individuals with AN may unintentionally limit gluten consumption as part of their calorie restriction, even before seeking medical care. This practice can reduce the symptoms of CD, potentially leading to diagnostic delays or inaccuracies because adequate gluten consumption is necessary before testing to trigger intestinal inflammation and its related symptoms [31]. Furthermore, individuals with CD often exhibit increased control around food, and the vigilance required to maintain dietary safety can lead to anxiety and obsessive thinking around food [32,33]. The maladaptive eating behaviors typical of EDs can intensify such control and anxiety, thus exacerbating the malabsorption issues in untreated CD, and worsening nutritional deficiencies and overall health outcomes.

Compared to the prevalence of EDs in individuals with CD, the prevalence of CD in individuals with EDs was lower. In the systematic review and meta analysis, the rate of CD in individuals with EDs was 0.90%, with subgroup meta-analysis revealing a rate of 1% in adults and 0.48% in children [13]. These findings showed the bidirectional association between EDs and CD, highlighting a potentially shared underlying mechanism.

## 4. Overlap Mechanisms for Developing Celiac Disease and Eating Disorders

**Figure 1:**
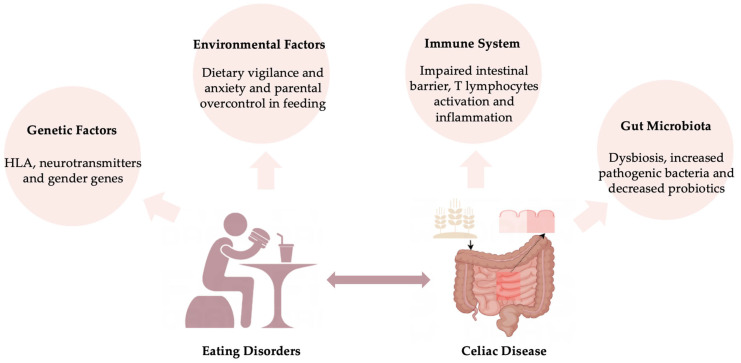
Overlap mechanisms for developing celiac disease and eating disorders.

Understanding the mechanisms that link CD to EDs is important in developing treatment and prevention strategies and being able to improve prognosis in individuals with CD. Current knowledge highlights influences such as genetic factors, environmental factors, immune system, and gut microbiome factors (Figure 1).

### 4.1. Genetic Factors

Both CD and EDs are genetically predisposed. The key genes in the pathogenesis of CD are primarily dependent on the HLA phenotype, specifically associated with HLA DQ2 and HLA DQ8 [34], either HLA DR3/DQ2 (mainly DQA1 *0501-DQB1 *0201, approximately 85% to 95%) or HLA DR4/DQ8 (DQA1 *0301-DQB1 *0302, about 5% to 15%), or a combination of both haplotypes [2,35]. The contribution of HLA genes to the heritability of CD is estimated to be between 30% and 50% [36]. Although CD susceptibility genes are mainly found in the HLA region, HLA genes are not the only genetic factors associated with CD. Studies identified 39 CD-associated non-HLA loci containing 115 distinct genes, such as cytotoxic T lymphocyte-associated antigen-4, interleukin (IL)2/IL21 gene, IL18 receptor helper protein gene, T-cell activation GTPase activating protein genes, and IL12A genes, which are highly associated with CD [37].

Genetic susceptibility to EDs is primarily linked to neurotransmitters, including serotonin (5-HT) and dopamine, as well as brain-derived neurotrophic factor. Additionally, estrogen is associated with ghrelin, agouti-related peptide, and other related genes [38]. It was found that people with anorexia expressed HLA-Bw16 more frequently than normal controls [39,40], while those with bulimia did not differ significantly from healthy controls in the frequency of any HLAA, -B, -C, or -DR antigens [41]. The biological hypothesis that HLA typing may cause EDs needs to be further studied.

In addition, studies have confirmed that there is a certain correlation between both conditions and gender. EDs are most common in adolescent girls and young adult women, while much less frequent in men. Lifetime prevalence rates for AN reach up to 4% in females compared to 0.3% in males. For BN, around 3% of females and over 1% of males are affected [42]. CD is also more common in women, and its prevalence is twice that of men; in adult CD individuals, the ratio of men to women is even 1:4 [16,43]. Several studies have indicated that CD is at least twice as prevalent in females compared to males [44]. This gender disparity suggests that genetic and hormonal factors may contribute to susceptibility in both conditions. Shared genetic loci or immune-related genes may partially explain the increased vulnerability among females.

### 4.2. Environmental Factors

Gluten contamination is the most common cause of ongoing gastrointestinal symptoms and intestinal inflammation in those with CD. Trace amounts of gluten in the diet can trigger disease [45]. Research suggests that the strict dietary vigilance required for managing CD may lead to a heightened state of anxiety and impact psychological well-being [46,47]. Increased expenses, limited availability of gluten-free options, challenges with dining out or traveling, social limitations due to dietary differences, and often lower taste quality of gluten-free foods can collectively reduce the quality of life for individuals with CD. Supporting this perspective, longitudinal studies have shown that negative emotions—such as anxiety and anger—are reliable predictors of various unhealthy eating patterns and are associated with an increased risk of clinically significant eating disorders, including AN and BN [48]. High anxiety plays a role in catastrophizing, and this recognition is thought to contribute to EDs in CD individuals [49]. Satherley et al. found that excessive control of diet or limited food choices may lead to the development of EDs in these people, even though most people with CD seem to be able to follow the GFD [50]. Research has identified hidden risk factors for EDs in the ways 53.3% of CD adolescents maintain a GFD, e.g., displaying stiffness, controlling behaviors, and especially in avoidance [51,52].

Parents play a critical role in managing dietary protocols for children with CD, ensuring adherence to prescribed nutritional guidelines, particularly given the early onset of the condition. Studies have shown that parents of children with CD often experience elevated levels of anxiety and depression, along with reduced quality of life [53,54]. This elevated parental anxiety can, in turn, impact the child’s mental health and may lead to overcontrol in feeding practices [55,56]. For instance, excessive control over a child’s feeding may limit their self-regulation for eating and appetite, such as self-control, as well as internal signals for hunger and fullness [57,58]. Also, children may seek autonomy by managing their eating habits and weight as a way of “anticontrol” to gain a sense of self-control, which may lead to excessive dietary restrictions or binge eating [59]. Childhood EDs are strongly associated with parents’ eating attitudes and habits, as well as negative comments about the weight of children [60]. Additionally, unhealthy family environment characteristics are also risk factors for children’s EDs, e.g., low flexibility, poor communication, conflict avoidance, and overprotectiveness [61]. These dynamics underscore the importance of fostering a balanced and supportive family environment to mitigate potential risks for disordered eating behaviors in children with CD.

### 4.3. Immune System

The intestinal epithelium, composed of a single-cell layer, serves as a crucial barrier safeguarding the intestine from external factors. Under normal physiological conditions, intestinal epithelial cells prevent the penetration of substances such as wheat gluten and gliadin. However, in pathological states, particularly in individuals with gastrointestinal conditions and mental disorders, the integrity of the tight junctions within the intestinal epithelial barrier is compromised [62,63]. This leads to an increased paracellular permeability, allowing external factors to cross the intestinal barrier. Consequently, the intestinal immune system is activated, further exacerbating the dysfunction of the epithelial barrier.

The pathogenesis of CD is closely related to the activation of T lymphocytes and the release of inflammatory mediators. Research has identified HLA DQ2/DQ8-specific T cells within mucosal lesions in individuals with CD. Due to incomplete digestion of gluten proteins by intestinal enzymes, peptide fragments are generated that bind to HLA, triggering T-cell infiltration into the intestinal mucosa. Activated CD4+ T cells promote cytokine-mediated inflammation, while infiltrating lymphocytes activate cytotoxic CD8+ T cells, which specifically target and damage inflamed intestinal epithelial cells, resulting in tissue injury [64,65]. Immune dysregulation is also evident in the context of EDs. For example, people with AN exhibit aberrant immune cell function, including an elevated CD4/CD8 ratio and impaired neutrophil chemotaxis, contributing to systemic inflammation [66]. This immune imbalance underscores one of the shared characteristics of the inflammatory mechanisms of both diseases.

Additionally, certain gluten peptides induce the expression of IL-15, CD83, CD25, and COX-2 in intestinal tissues, further increasing intestinal permeability. This immune activation also stimulates B cells, leading to the production of autoantibodies against tissue transglutaminase and other endogenous antigens [67,68]. The resulting immune cascade causes significant inflammation of the intestinal mucosa, villous atrophy, and increased intestinal permeability. Inflammatory responses are also evident in EDs. For example, people with AN exhibit increased levels of proinflammatory cytokines, such as IL-17, IL-6, and TNF-α, along with a higher CD4/CD8 ratio and reduced neutrophil chemotaxis [69,70,71]. Immune dysfunction also appears to be a significant factor in bulimic syndromes. Individuals with BN exhibit a primary deficiency in T-cell levels, accompanied by elevated concentrations of high-sensitivity C-reactive protein (hs-CRP), a marker of systemic inflammation [72,73]. Notably, studies have found that adolescents with loss-of-control eating behaviors also display heightened hs-CRP levels, suggesting a link between inflammatory processes and disordered eating patterns in BN [72,74].

### 4.4. Gut Microbiota

The gut microbiota is closely associated with the “brain–gut axis”, a bidirectional communication network linking the brain’s emotional and cognitive centers with the gastrointestinal tract [75]. Recent research suggests that gut microbiota dysbiosis may play a role in the pathogenesis of both CD and EDs [76,77]. The gut microbiota is essential for maintaining immune homeostasis, and changes in its composition and metabolite production can alter immune cell responses, contributing to immune-mediated inflammatory disorders, such as inflammatory bowel diseases, diabetes, and rheumatoid arthritis [78]. Beneficial microbiota may ameliorate intestinal damage by modulating intestinal permeability and influencing cytokines production, balancing pro-inflammatory and anti-inflammatory signals [76].

Furthermore, studies have uncovered molecular signaling between gut microbes and the central nervous system, with gut inflammation in CD reducing levels of essential amino acids needed for serotonin synthesis [79]. This reduction in brain serotonin is implicated in various ED-related behaviors, including binge eating, impulsivity, and difficulties with mood regulation. Both CD and EDs exhibit symptoms that fluctuate with body weight. Alterations in weight, common to both conditions, may be influenced by the regulation of the gut microbiota, which modulates epigenetic modifications, metabolic pathways, and the microbiota–gut–brain axis [80]. Certain probiotic strains, such as *Lactobacillus plantarum* and *Bifidobacterium animalis*, have shown promise in alleviating gastrointestinal distress, highlighting their potential therapeutic role [81]. Moreover, *Faecalibacterium prausnitzii*, known for its anxiolytic and antidepressant-like effects in animal models, has emerged as a potential next-generation probiotic for managing both gut inflammation and neuropsychiatric symptoms [82]. Given the overlapping features of CD and EDs, using probiotics as adjunctive therapy could represent an innovative approach to managing these conditions, supporting both gastrointestinal health and mental well-being.

## 5. Clinical and Research Implications

The bidirectional relationship between EDs and CD presents challenges for clinical management and research. The complex interplay between these conditions necessitates a comprehensive approach to screening, treatment, and ongoing management, highlighting several potential areas to support the quality of life and well-being of individuals.

### 5.1. Tailored Screening and Monitoring Approaches

Considering the presence of silent CD and its frequent comorbidity with disordered eating, it is essential to adopt a tailored approach to screen and monitor, taking into account the individual risks of both conditions. Avila et al. [83] recommended that adolescents and young adults presenting with symptoms typical of EDs should be screened for CD and inflammatory bowel disease, especially when they exhibit unexplained abdominal pain. When suspected, the initial CD-screening step is to check specific celiac serological tests, including IgA anti-tissue transglutaminase antibody (IgA anti-tTG Ab), IgA endomysial antibody (IgA EMA), and IgG deamidated gliadin peptide antibody (IgG anti-DGP Ab). Currently, specific serological tests are the first-line method for screening CD, but a small intestinal biopsy remains the definitive “gold standard” for diagnosis [84,85]. However, many studies believe that universal screening for CD in all individuals suspected of having an eating disorder is not recommended and non-cost-effective due to the relative rarity of CD in such a population [86,87,88]. This necessitates further research to identify cost-effective strategies that can pinpoint high-risk individuals more accurately.

By contrast, screening tools specifically designed for detecting EDs in individuals with CD are relatively economical and easy to conduct, considering the main screening tools for EDs are questionnaires, such as the Eating Disorder Examination Questionnaire and Eating Disorder Inventory [89,90]. Also, screening is essential because earlier diagnosis and treatment of EDs is associated with an improved prognosis [91,92]. However, existing ED screening questionnaires are mainly designed for general populations and may not effectively address the nuanced interplay between the management of chronic CD and specific disordered eating behaviors, including gluten fear and avoidance. One good example is the Diabetes Eating Problem Survey—Revised, which has been widely used to study people with type 1 diabetes mellitus who are at risk of developing or have already developed diabulimia [93]. This scale contains evaluations of diabetes-specific eating behaviors, such as insulin omission or restriction, which general assessment tools do not cover [94]. The development of celiac-disease-specific disordered eating screening tools could allow for the earlier and more accurate detection of maladaptive behaviors.

### 5.2. Enhancing Clinical Practice Through Multidisciplinary Care

The “double whammy” effect, as described by Sawyer et al. [95], refers to the doubly disadvantaged individuals with chronic health conditions like CD, who also engage in disordered eating behaviors. These individuals not only face the physiological complications associated with their chronic condition, but also experience exacerbated health outcomes due to EDs. Addressing both the psychological aspects of CD and the physiological impacts of EDs in tandem can improve patient outcomes. A multidisciplinary team approach, considered the best practice for treating EDs [96], is equally valuable in managing CD. Managing a GFD requires careful attention to food and gluten avoidance, some individuals with CD may develop intense anxiety around gluten exposure, and sometimes unrealistic beliefs about dietary and lifestyle management [52]. Individuals’ well-being could be impaired even when they seem to adhere well to a GFD clinically, due to maladaptive coping strategies linked to dietary restrictions [52].

Healthcare providers’ involvement is a critical factor in alleviating gluten-related distress. Post-diagnosis clinical support and education are crucial for empowering individuals with the knowledge and confidence to manage their GFD, and, when necessary, referrals to psychological support services can help in identifying and addressing impaired well-being and maladaptive thoughts and behaviors related to food [25,52]. Although there are no evidence-based interventions specifically targeting maladaptive attitudes and dietary behaviors in CD, the growing body of research on psychological treatments for other gastrointestinal conditions, such as irritable bowel syndrome, may provide a good example for developing similar strategies for celiac individuals [97].

### 5.3. Prioritizing Future Research to Understand and Address Complex Interactions

Evidence indicates that there is a bidirectional relationship between CD and EDs. Although the factors involved in the association are just beginning to be unveiled, many questions remain. For example, why is this relationship asymmetrical, with CD individuals having a higher likelihood of developing EDs than ED individuals developing CD? What are the biopsychosocial mechanisms that underlie the relationship between CD and EDs? Do these act independently or in synergy to promote the conditions? What central nervous system pathways are affected by CD pathology in ways that promote EDs? Are there specific changes that predispose some individuals to EDs, or are certain changes common across many EDs individuals?

Exploring how these conditions influence one another can lead to better therapeutic strategies that address the root causes rather than just the symptoms. This includes studying the impact of gluten exposure and the psychological stressors that may trigger or worsen eating disorder symptoms in individuals with CD. Investigations into how CD serves as a model for understanding EDs could further elucidate the complex interactions between chronic physical illnesses and psychological health. Research into effective psychosocial interventions that incorporate coping mechanisms for dealing with chronic illness can improve the quality of life and health outcomes for these individuals.

## 6. Conclusions

CD and EDs share a significant bidirectional association. Both conditions present with similar symptoms and can be misdiagnosed, but they can also coexist, making management challenging. By refining screening and monitoring strategies, enhancing clinical practice through integrated care, and prioritizing targeted research initiatives, healthcare providers can better support individuals affected by CD and EDs.

## Figures and Tables

**Table 1:** Clinical manifestations and treatments for celiac diseases and eating disorders.

Disease Type	Clinical Manifestations	Clinical Treatments
**Celiac diseases**	**Gastrointestinal symptoms**: constipation, diarrhea, bloating and gas, abdominal pain and distension, nausea and vomiting **Symptoms related to malabsorption**: weight loss, growth failure, anemia, osteoporosis **Others**: skin rash, fatigue, headache, neurological symptoms (migraine, ataxia)	**Gluten-Free diet:** strictly avoid grains containing gluten, such as wheat, barley, oats, and rye along with their derivatives. Safe alternatives include rice, corn, millet, buckwheat, and others
**Eating disorders**	**Anorexia nervosa**: characterized by an intense fear of weight gain and self-imposed starvation leading to extremely low body weight	**Nutrition therapy**: often involves monitored renourishment, eating habits development, and mindful eating **Psychological therapy**: cognitive behavioral therapy is recommended for various eating disorders. Family therapy, dialectical behavior therapy, and interpersonal psychotherapy also have evidence supporting their effectiveness **Medication treatment**: antidepressants, such as fluoxetine, are recommended for the treatment of bulimia nervosa and binge-eating disorder. Lisdexamfetamine also has supporting evidence for treating binge-eating disorder
	**Bulimia nervosa**: manifested as episodes of binge eating followed by compensatory behaviors such as self-induced vomiting and excessive exercise to prevent weight gain	
	**Binge-eating disorder**: characterized by recurrent episodes of binge eating without subsequent compensatory behaviors	
**Shared features of the two conditions**	**Gastrointestinal symptoms**: constipation, abdominal pain and distension, nausea and vomiting, bloating and gas **Symptoms related to malabsorption**: weight loss, growth failure, anemia, osteoporosis **Others**: fatigue, headache, neurological symptoms	Both conditions involve dietary management and recommend psychological interventions to address eating-related cognitive and emotional burdens

## Data Availability

The original contributions presented in the study are included in the article, further inquiries can be directed to the corresponding authors.
